# Exogenous Trehalose Treatment Enhances the Activities of Defense-Related Enzymes and Triggers Resistance against Downy Mildew Disease of Pearl Millet

**DOI:** 10.3389/fpls.2016.01593

**Published:** 2016-11-15

**Authors:** Sharathchandra R. Govind, Sudisha Jogaiah, Mostafa Abdelrahman, Hunthrike S. Shetty, Lam-Son P. Tran

**Affiliations:** ^1^Department of Studies and Research in Environmental Science, Centre for Bioinformation, Tumkur UniversityTumkur, India; ^2^Plant Healthcare and Diagnostic Center, Department of Studies in Biotechnology and Microbiology, Karnatak UniversityDharwad, India; ^3^Graduate School of Life Sciences, Tohoku UniversitySendai, Japan; ^4^Department of Botany, Faculty of Science, Aswan UniversityAswan, Egypt; ^5^Downy Mildew Research Laboratory, Department of Studies in Biotechnology, University of MysoreMysore, India; ^6^Plant Abiotic Stress Research Group & Faculty of Applied Sciences, Ton Duc Thang UniversityHo Chi Minh City, Vietnam; ^7^Signaling Pathway Research Unit, RIKEN Center for Sustainable Resource ScienceYokohama, Japan

**Keywords:** pearl millet, downy mildew, induction of resistance, trehalose, stress responses

## Abstract

In recent years, diverse physiological functions of various sugars are the subject of investigations. Their roles in signal transduction in plant responses to adverse biotic and abiotic stress conditions have become apparent, and growing scientific evidence has indicated that disaccharides like sucrose and trehalose mediate plant defense responses in similar way as those induced by elicitors against the pathogens. Trehalose is a well-known metabolic osmoregulator, stress-protectant and non-reducing disaccharide existing in a variety of organisms, including fungi, bacteria, and plants. Commercially procured trehalose was applied to seeds of susceptible pearl millet (*Pennisetum glaucum*) cultivar “HB3,” and tested for its ability to reduce downy mildew disease incidence by induction of resistance. Seed treatment with trehalose at 200 mM for 9 h recorded 70.25% downy mildew disease protection, followed by those with 100 and 50 mM trehalose which offered 64.35 and 52.55% defense, respectively, under greenhouse conditions. Furthermore, under field conditions treatment with 200 mM trehalose for 9 h recorded 67.25% downy mildew disease protection, and reduced the disease severity to 32.75% when compared with untreated control which displayed 90% of disease severity. Trehalose did not affect either sporangial formation or zoospore release from sporangia, indicating that the reduction in disease incidence was not due to direct inhibition but rather through induction of resistance responses in the host. Additionally, trehalose was shown to enhance the levels of polyphenol oxidase, phenylalanine ammonia lyase, and peroxidase, which are known as markers of both biotic and abiotic stress responses. Our study shows that osmoregulators like trehalose could be used to protect plants against pathogen attacks by seed treatment, thus offering dual benefits of biotic and abiotic stress tolerance.

## Introduction

Increasing crop production in the reducing fertile land cover is one of the major focuses in modern agriculture and green innovation. Climate resilient crops like pearl millet are therefore of prime importance for sustainable food production. Downy mildew disease caused by the oomycete *Sclerospora graminicola* is one of the most destructive diseases in pearl millet (*Pennisetum glaucum*), which affects its yield, and as a consequence income of resource-poor farmers (Nutsugah et al., 2002). The classical control of this disease is mainly dependent on host resistance genotypes and fungicides (Jogaiah et al., 2007; Murali et al., 2013). However, because *S. graminicola* occupies distinct physiological and phylogenetic niche, most of the disease control measures have failed to obtain a durable downy mildew disease resistance (Jogaiah et al., 2014). Moreover, pesticides have many concerns with regards to human health, environmental degradation, and evolution of the pathogens that have enhanced levels of resistance to pesticides. Thus, research for the development of new strategies for sustainable pearl millet production is the need of the hour (Trouvelot et al., 2014).

Application of molecules that trigger plant resistance against pathogens through the elicitation of defense responses, have emerged as an effective and eco-friendly new approach for managing crop diseases (Garcia-Mier et al., 2013; Pel and Pieterse, 2013; Pushpalatha et al., 2013). The applications of such elicitors or resistance inducers at low doses are not only effective against a broad spectrum of pathogens in various plants, but also enhance crop yield without asserting selective pressures on pathogen populations (Nandeeshkumar et al., 2008; Rasul et al., 2012; Pushpalatha et al., 2013; Aranega-Bou et al., 2014; Katiyar et al., 2015). A number of such inducers have been tested against pearl millet downy mildew disease, including oligosaccharides supplemented with mannitol, *N*-acetylchitooligosaccharides, β-aminobutyric acid, and 3,5-dichloroanthranilic acid (Sharathchandra et al., 2004; Nandini et al., 2013; Shailasree and Melvin, 2015; Lavanya and Amruthesh, 2016). However, previous attempts with various classes of inducing agents have resulted in identification of only a few bioagents for durable downy mildew disease resistance (Jogaiah et al., 2010; Raj et al., 2012; Murali et al., 2013). Thus, the present situation demands a constant and concerted effort in order to create a panel of effective inducers of downy mildew disease resistance in pearl millet.

Sugars, such as trehalose, sucrose, fructose, and glucose, are involved in many signaling and metabolic pathways in plants (Smeekens et al., 2010; Trouvelot et al., 2014). Trehalose (α-D-glucopyranosyl-[1–1]-α–D-glucopyranoside), a non-reducing disaccharide comprising of two units of glucose, is widely spread in plants, fungi and bacteria (Elbein et al., 2003). In plants, trehalose serves as a signaling molecule (Paul et al., 2008; Kumar et al., 2013), acting as a regulator of various biological process, including plant growth, development, senescence (Paul et al., 2008; Wingler et al., 2012), as well as plant responses to abiotic and biotic stresses (Singh et al., 2011; Krasensky and Jonak, 2012; Lyu et al., 2013; Delorge et al., 2014; Mostofa et al., 2015). With regard to biotic stress resistance; for instance, exogenous application of trehalose has been shown to enhance *Arabidopsis thaliana* defense against green peach aphid (Singh et al., 2011), as well as resistance of wheat (*Triticum aestivum* L.) against powdery mildew caused by *Blumeria graminis* (Reignault et al., 2001; Muchembled et al., 2006; Tayeh et al., 2014).

Given the potential of trehalose in stress resistance, the present study was carried out to evaluate the efficacy of trehalose in triggering seed germination, and growth, as well as disease resistance of pearl millet plants against downy mildew disease through seed treatment under both greenhouse and field conditions. Additionally, we also examined the activities of polyphenol oxidase (PPO), phenylalanine ammonia lyase (PAL), and peroxidase (POX) as a means to gain an insight into the defense mechanisms mediated by trehalose in pearl millet plants to enhance their resistance against *S. graminicola*.

## Materials and Methods

### Chemicals and Reagents

Trehalose (99.5% pure) was purchased from Sigma-Aldrich Co. (St. Louis, MO, USA). Millipore-grade water was used in all experiments.

### Plant Materials

Pearl millet (*Pennisetum glaucum*) “HB3” and “1P18192” are highly susceptible and resistant cultivars, respectively, to downy mildew (Jogaiah et al., 2016). “HB3” seeds were received from All India Coordinated Pearl Millet Improvement Project (AICPMIP), Jodhpur, India, whereas “1P18192” seeds were obtained from the International Crops Research Institute for the Semi-Arid Tropics (ICRISAT), Patancheru, India.

### Pathogen and Inoculum Preparation

*Sclerospora graminicola* was isolated from “HB3” and maintained under greenhouse conditions as previously described (Jogaiah et al., 2016). The concentration of zoospores released from sporangia was adjusted to 4 × 10^4^ zoospores mL^-1^ with sterile distilled water (SDW) using a haemocytometer.

### Seed Germination and Seedling Vigor

“HB3” seeds were surface-sterilized according to Jogaiah et al. (2016), before trehalose treatment. Two grams of seeds were soaked in 20 mL of various concentrations (25, 50, 100, and 200 mM) of trehalose or SDW (control), incubated at 25 + 2°C for 3, 6, and 9 h, and then blot-dried. Treated seeds were subjected to a germination assay as described by Sudisha et al. (2010). Seedling vigor of 7-day-old seedlings was examined according to Murali et al. (2013). Both the seed germination and seedling vigor experiments were conducted with four replicates of 25 seeds per treatment, and were repeated twice.

### *Sclerospora graminicola* Sporangial Development and Zoospore Release from Sporangia

For evaluation of sporangial development, infected susceptible “HB3” leaves were collected, cleaned and cut into pieces of 1 cm^2^ size. Subsequently, the leaf samples were treated with various concentrations of trehalose (25, 50, 100, and 200 mM) and SDW for 1 h, and then incubated in a moist chamber for overnight sporulation at 20°C and 95% relative humidity. Sporulated sporangia from each treatment were collected in 1 mL of SDW, and sporangial development was assessed using a haemocytometer. The sporangial formation assay was carried out in four replicates with three leaf pieces per replicate, and the total number of sporangia was determined as previously described (Jogaiah et al., 2007).

To assess the influence of the different concentrations of trehalose on zoospore releases, 100 μL sporangial suspension (4 × 10^4^ sporangia mL^-1^) was harvested from the susceptible “HB3” leaves, mixed with 100 μL of trehalose (25, 50, 100, and 200 mM) or SDW, and then placed on cavity block glass slide. The slides were incubated at 22 + 2°C in dark for 30 min, and the percentage of zoospore release was determined according to Jogaiah et al. (2007). For the zoospore release assay, the treatment consisted of four replications with three cavity block glass slides per replicate.

### Assay for Downy Mildew Disease Protection under Greenhouse Conditions

“HB3” seeds were treated with various concentrations of trehalose or SDW for different durations as described above, and sown in earthen pots. Two-day-old seedlings were inoculated with SDW or zoospores of *S. graminicola* at a concentration of 4 × 10^4^ zoospores mL^-1^ according to Jogaiah et al. (2014). Apron 35SD (6 g Kg^-1^ seeds) was used as a standard chemical for positive control (Sudisha et al., 2009). The disease severity percentage was recorded after 30 days after treatments. The experiment was performed in four replicates, with 10 seedlings per replicate, and repeated twice.

### Assay for Downy Mildew Disease Resistance under Field Conditions

“HB3” seeds, which were treated with different concentrations of trehalose or SDW and incubated at 25 + 2°C for 9 h as described above, were used for this experiment. Trials were performed at the Downy Mildew Sick Plot, University of Mysore, Mysore, India (N26_18°, E73_30°, 817 m altitude, red loam soil) during Kharif season. The plot has been naturally infected with zoospores of *S. graminicola* for more than three decades, and these zoospores were used as a primary inoculum. Additional *S. graminicola* inoculum was supplied by the infected rows (pathogen source), which were seeded 21 days before the test rows (treated seeds) were sowed, as described by Williams (1984). Each treatment had four replications, and regular agronomic practices were followed to grow the plants with equal irrigation. Thinning was carried out after 15 days of sowing to maintain equal number of plants (25 plants) per row and the same distance between the plants. The plants in the test rows were examined for downy mildew symptom development at days 30th and 60th after sowing, and scored as diseased when they showed the typical downy mildew symptoms, which are as follows: sporulation of *S. graminicola* on the abaxial leaf surface, stunted growth, chlorosis, and malformation of the ear heads. The data were consolidated after 60 days of sowing. Percentage of disease protection was computed using the following formula:

$$\%\;\mathrm{protection}=\frac{\begin{array}{c} 100\times (percent downy mildew of untreated\\ plants-percent downy mildew of treated plants) \end{array}}{percent downy mildew of untreated plants}$$

In addition, severity of the disease was also recorded on a 1–5 rating scale according to the procedure of Singh et al. (1997). The experiments were conducted in four replicates with 100 seedlings each, and repeated twice. Apron 35SD (6 g Kg^-1^ seeds) was used as a standard chemical for positive control in the field experiment (Sudisha et al., 2009).

### Analyses of Defense-Responsive Enzymes

#### Inoculum Preparation, Inoculation, and Sampling

To examine the comparative effects of trehalose treatment on the defense-responsive enzymes, the following scheme of inoculation and sampling were followed:

(1) Susceptible “HB3” seeds were primed with 200 mM trehalose for 9 h and raised on three layers of moist blotter disks in petri plates for 2 days. Thereafter, the emerging seedlings were inoculated with *S. graminicola* (4 × 10^4^ zoospores mL^-1^) by root-dip inoculation. This set of treatment was termed as inducer-treated and pathogen-inoculated (ITPI).
(2) Susceptible “HB3” seeds were primed with 200 mM trehalose for 9 h and raised on three layers of moist blotter disks in petri plates for 2 days. Thereafter, the emerging seedlings were inoculated with SDW by root-dip inoculation for comparison. This set of treatment was termed as inducer-treated control (ITC).
(3) In order to compare the innate resistance with that of the inducer treatment, susceptible “HB3” seeds were treated with SDW for 9 h and raised on three layers of moist blotter disks in petri plates for 2 days. Thereafter, the emerging seedlings were inoculated with *S. graminicola* (4 × 10^4^ zoospores mL^-1^) by root-dip inoculation. This set of treatment was termed as non-induced and pathogen-inoculated (NIPI).
(4) Susceptible “HB3” seeds were treated with SDW for 9 h and raised on three layers of moist blotter disks in petri plates for 2 days. Thereafter, the emerging seedlings were inoculated with SDW by root-dip inoculation for comparison. This set of treatment was termed as non-induced control (NIC).
(5) In order to compare the innate resistance of known resistant genotype with that of the inducer effect on the susceptible “HB3” genotype, seeds of resistant “IP18192” genotype were raised on three layers of moist blotter disks in petri plates for 2 days. Thereafter, the emerging seedlings were inoculated with *S. graminicola* (4 × 10^4^ zoospores mL^-1^) by root-dip inoculation. This set of treatment was called resistant-inoculated (RI).
(6) For control, resistant “IP18192” seeds were raised on three layers of moist blotter disks placed in petri plates for 2 days. Later, the emerging seedlings were inoculated with SDW by root-dip inoculation. This set of treatment was termed as resistant control (RC).

Samples (one gram fresh weight per sample) were collected from all the treatments in a time course of 3, 6, 12, 24, 48, and 72 h post-inoculation (hpi), dipped in liquid nitrogen for 5 min and immediately stored at -80°C until use for enzyme assays.

#### Assays for PPO, PAL, and POX Activities

PPO (EC 1.14.18.1) activity was assayed using a Hitachi (U2000, Tokyo, Japan) UV-visible spectrophotometer according to the method of Arora and Bajaj (1985). The reaction mixture (3 mL) comprised 25 mM citrate phosphate buffer (pH 6.4), 40 μL of the enzyme extract, and 5 mM proline in 20 mM of pyrocatechol (1,2-dihydroxybenzene) substrate (Arora and Bajaj, 1985). The PPO activities were expressed as the change in Δ515 mg^-1^ protein min^-1^.

Phenylalanine ammonia lyase (EC 4.3.1.5) activity was quantified in a reaction mixture (3 mL) consisting of 50 mM phenylalanine in 100 mM sodium borate buffer (pH 8.8), 500 μL of the enzyme extract, and 25 mM sodium borate buffer (pH 8.8) in 32 mM β-mercaptoethanol as described by (Geetha et al., 2005). The activity was measured using a spectrophotometer at 290 nm and expressed in mol t-Cinnamic acid mg^-1^ protein h^-1^.

Peroxidase (EC 1.11.1.8) activity was determined using guaiacol as hydrogen donor as previously reported by Hammerschmidt et al. (1982). Five microliter of the enzyme extract was added to 3 mL reaction mixture of 0.25% (v/v) guaiacol in 100 mM potassium phosphate buffer (pH 6.0) and 100 mM H_2_O_2_. The absorbance of the product was measured spectrophotometrically at 470 nm, and used in calculation of enzyme activity expressed in mg^-1^ protein min^-1^.

### Statistical Analysis

Data obtained were subjected to a one-way analysis of variance (ANOVA). Significant differences among the treatments (*P ≤* 0.05) obtained by Tukey’s honestly significant difference (HSD) *post hoc* test using SPSS software (SPSS20.0, SPSS Inc., USA) are depicted in the Figures by various letters. Values shown in the Figures are the means ± standard errors (SEs) of four independent replicates.

## Results

### Effects of Seed Treatment with Trehalose on Seed Germination and Seedling Vigor

**FIGURE 1:**
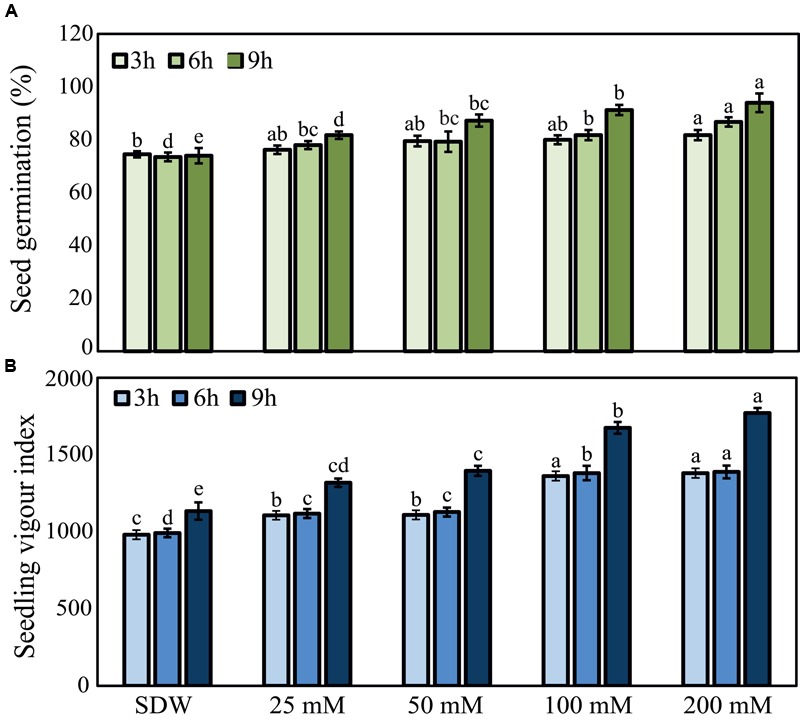
**Effects of trehalose on peal millet susceptible “HB3” seed germination percentage and seedling vigor index. (A)** Seed germination percentage and **(B)** seedling vigor index of “HB3” susceptible pearl millet after seed-soaking in various concentrations of trehalose for 3, 6, and 9 h in comparison with sterile distilled water (SDW) control treatment. Values are means ± standard errors (SEs) of four independent replicates (*n* = 4, where each replicate represents 25 seeds). Bars followed by different letters are significantly different according to a Tukey’s honestly significant difference *post hoc* test (*P* ≤ 0.05).

Seed germination percentage and seedling vigor index were recorded for all the treatments with various concentrations of trehalose (**Figure 1**). In general, long-term 9 h treatment of pearl millet seeds with trehalose exhibited higher effects than 3 or 6 h treatments, and the higher the concentration of trehalose was used the significantly higher effect was observed. “HB3” seeds treated with 200 mM trehalose recorded maximum germination percentage of 94.0% and seedling vigor index of 1771.0 at 9 h, while SDW-treated control seeds recorded significantly lower values with 74.0% germination percentage and 1134.75 seedling vigor index (**Figure 1**). These data demonstrated the positive effects of seed-priming with trehalose on seed germination as well as growth of pearl millet plants at early stages.

### Effects of Trehalose on *S. graminicola* Sporangial Development and Zoospore Release from Sporangia

**FIGURE 2:**
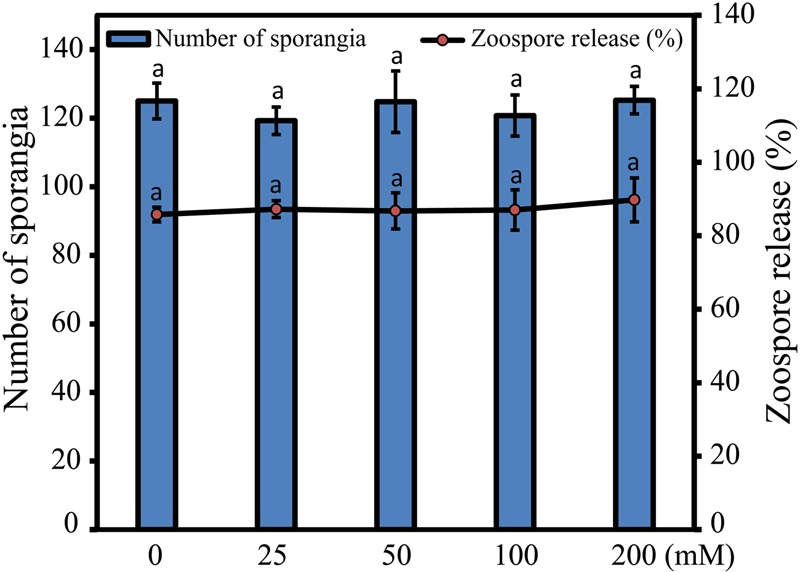
**Effects of trehalose on the *Sclerospora graminicola* sporangia formation and percentage of zoospore release from sporangia.** Primary axis indicates total number of sporangia, while secondary axis shows percentage of zoospore release from sporangia after treatment with various concentrations of trehalose in comparison with sterile distilled water control treatment (0 mM). Values are means ± standard errors (SEs) of four independent replicates (*n* = 4, where each replicate represents three leaf pieces in case of sporangial formation or three cavity block glass slides in case of zoospore release). Bars followed by same letters are insignificantly different according to a Tukey’s honestly significant difference *post hoc* test (*P* ≤ 0.05).

In order to test whether *S. graminicola* sporangia development was affected by trehalose treatment, infected leaves of “HB3” were treated with different concentrations of trehalose and SDW, and the comparative effects of trehalose on *in vitro* formation of sporangia per cm^2^ and zoospore release were microscopically studied. Our data indicated that there was no significant difference between trehalose and SDW treatments for either *in vitro* formation of sporangia or zoospore release (**Figure 2**). This finding suggested that under our experimental conditions, trehalose treatment did not have significant inhibition effects on *S. graminicola* sporangia development.

### Effects of Pearl Millet “HB3” Seed-Priming with Trehalose on Downy Mildew Disease Incidence under Greenhouse Conditions

**FIGURE 3:**
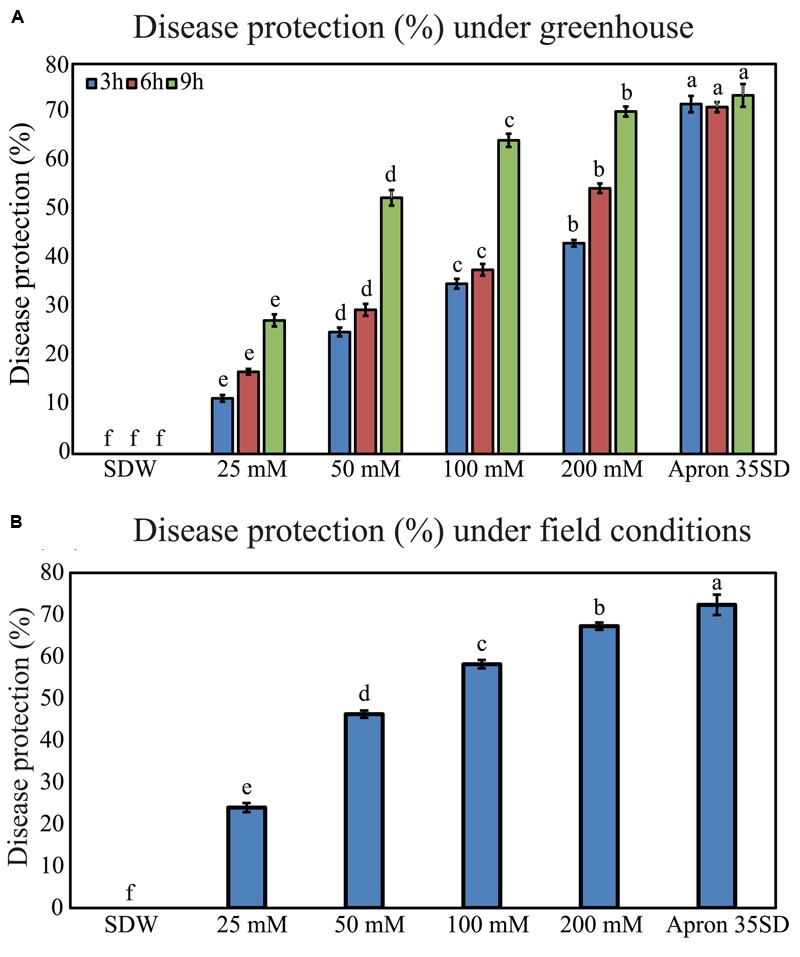
**Downy mildew disease protection rates in pearl millet susceptible “HB3” under greenhouse and field conditions following seed-priming with trehalose. (A)** Disease protection percentage under greenhouse and **(B)** under field conditions of the pearl millet susceptible “HB3” plants raised from seeds treated with various concentrations of trehalose (25, 50, 100, and 200 mM) and Apron 35SD in comparison with sterile distilled water (SDW) control treatment. Values are means ± standard errors (SEs) of four independent replicates (*n* = 4, where each replicate represents 25 plants). Different letters indicate significant differences according to a Tukey’s honestly significant difference *post hoc* test (*P* ≤ 0.05).

In the next line of our study, we examined the effects of seed-priming with trehalose on inhibition of downy mildew disease under greenhouse conditions. Pearl millet susceptible “HB3” seeds were pretreated with various concentrations of trehalose exhibited improved downy mildew disease resistance in duration- and dose-dependent manner. “HB3” seeds treated with 200 mM trehalose provided disease protection of 43.22, 54.50, and 70.25% at 3, 6, and 9 h, respectively; and thus, 200 mM of trehalose was found to be the most effective among all the concentrations tested. Treatment with 25 mM trehalose offered the lowest disease protection rates of 11.42, 16.85, and 27.40% at 3, 6, and 9 h, respectively (**Figure 3A**). More importantly, the disease protection efficiency of 200 mM trehalose treatment was on par with the Apron 35SD, which recorded 73.55% disease protection rate at 9 h. Plants generated from SDW-treated seeds were completely susceptible to *S. graminicola* infection (**Figure 3A**).

### Effects of Pearl Millet “HB3” Seed-Priming with Trehalose on Downy Mildew Disease Incidence and Severity under Field Conditions

We were then interested in testing the effects of seed treatment with trehalose on protection of pearl millet plants against downy mildew disease under field conditions, which is a pre-requisite for potential commercial use of trehalose. In our experimental design, field evaluation was carried out with pearl millet plants grown from seeds pretreated with 25, 50, 100, and 200 mM trehalose for 9 h, because the 9 h duration of treatment was found to be the most effective in decreasing the downy mildew disease incidence under greenhouse conditions (**Figure 3A**). Consistent with the data obtained from the greenhouse experiment, treatment with 200 mM trehalose exhibited the highest downy mildew disease protection under field conditions in comparison with lower concentrations (**Figure 3B**). Seed-priming with 25, 50, 100, and 200 mM trehalose for 9 h resulted in 23.97, 46.27, 58.20, and 67.25% downy mildew protection rates, respectively. In addition, seed treatment with 200 mM trehalose for 9 h offered protection on par with Apron 35SD at 72.35%. However, SDW exhibited no resistance to downy mildew disease under tested filed conditions (**Figure 3B**).

### Activities of Defense-Responsive Enzymes

**FIGURE 4:**
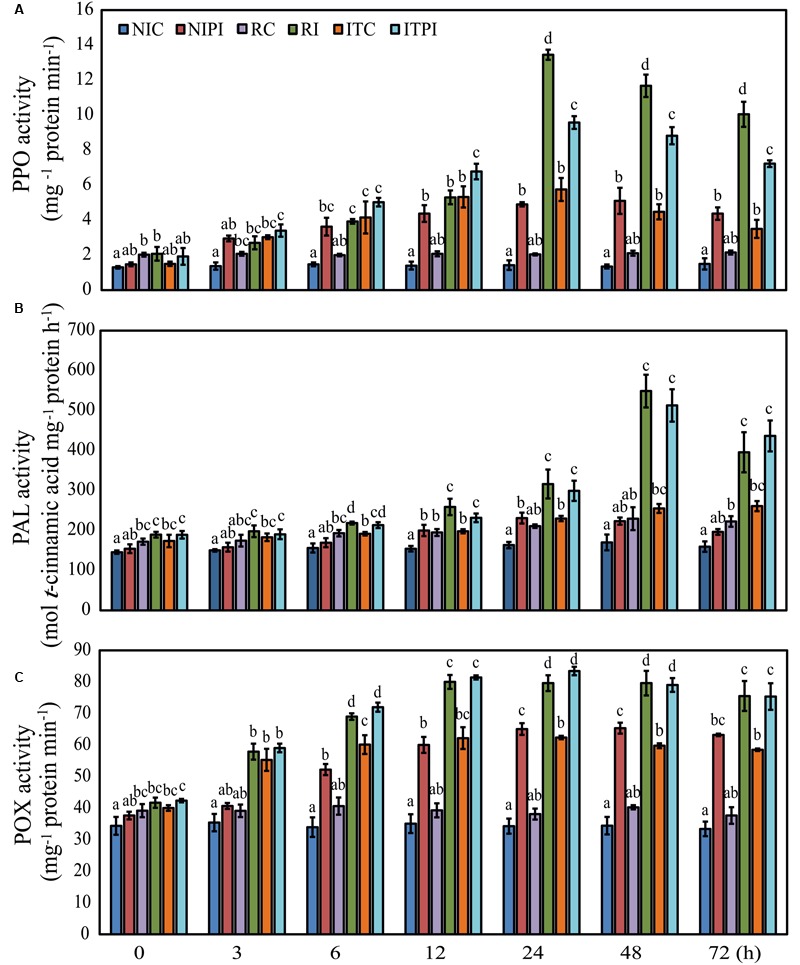
**Time course analysis of the polyphenol peroxidase (PPO), phenylalanine ammonia lyase (PAL), and peroxidase (POX) activities in 2-day-old pearl millet seedlings.** Activities of **(A)** PPO, **(B)** PAL, and **(C)** POX enzymes in 2-day-old susceptible “HB3” seedlings grown from seeds primed with sterile distilled water (SDW) or 200 mM trehalose for 9 h followed by root-dip inoculation with *Sclerospora graminicola* for various time periods (0, 3, 6, 12, 24, 48, and 72 h). “IP18192” seedlings grown from seeds primed with SDW for 9 h followed by root-dip inoculation with *S. graminicola* served as a positive control. Values are means ± standard errors (SEs) of four independent replicates (*n* = 4). Bars followed by different letters are significantly different according to a Tukey’s honestly significant difference *post hoc* test (*P* ≤ 0.05). Non-induced control (NIC), pearl millet susceptible “HB3” seedlings raised from seeds treated with SDW for 9 h, and thereafter challenged with SDW; NIPI, pearl millet susceptible “HB3” seedlings raised from seeds treated with SDW for 9 h and later challenged with *S. graminicola*; resistant control (RC), pearl millet resistant “IP18192” seedlings raised from seeds treated with SDW for 9 h and later challenged with SDW; resistant-inoculated (RI), pearl millet resistant “IP18192” seedlings raised from seeds treated with SDW for 9 h and later challenged with *S. graminicola;* ITC, pearl millet susceptible “HB3” seedlings raised from seeds treated with 200 mM trehalose for 9 h and later challenged with SDW; inducer-treated and pathogen-inoculated (ITPI), pearl millet susceptible “HB3” seedlings raised from seeds treated with 200 mM trehalose for 9 h and later challenged with *S. graminicola.*

Induction of resistance in host plants is known to be accompanied by an increase in defense-related enzymes, including PPO, PAL, and POX (Reignault et al., 2001; Nandeeshkumar et al., 2008; Jogaiah et al., 2013; Babu et al., 2015). Therefore, we have tested the ability of seed-priming with trehalose in inducing PPO, PAL, and POX using the experimental design described in detail in “Materials and Methods.” The NIC and RC samples showed a significant difference (*P* ≤ 0.05) from each other in terms of PPO activities. PPO activities of RC were 1.35- to 1.56-fold higher than those of NIC at different time intervals (**Figure 4A**). However, the ITC seedlings showed a significant increase in PPO activity with a maximum of 4.03-fold increase (*P* ≤ 0.05) at 24 h post treatment relative to that of NIC plants (**Figure 4A**), suggesting that trehalose could trigger induction of PPO even in the absence of pathogen. Furthermore, trehalose induced PPO activities in ITC in a duration manner, with maximum activity at 24 h followed by a steady decrease (Supplementary Figure S1A). Our data also showed that pathogen-inoculation resulted in a remarkable increase of upto 1.97-fold (*P* ≤ 0.05) in PPO activity at 24 hpi in ITPI plants when compared with that of ITC (Supplementary Figure S1A). A 6.59-fold increase (*P* ≤ 0.05) of PPO activity in RI plants relative to RC at 24 hpi was observed (**Figure 4A**). Additionally, our results clearly demonstrated that ITC plants recorded 2.81-fold (*P* ≤ 0.05) increase, when compared with RC plants at 24 hpi (Supplementary Figure S1A). Moreover, in the pathogen-inoculated ITPI plants the highest increase of 1.28-fold (*P* ≤ 0.05) in PPO activity was recorded at 6 hpi in comparison with the RI plants, with a steady decrease after 24 hpi (Supplementary Figure S1A).

Patterns of PAL activities observed were found to be similar to those of the PPO activities (**Figure 4B**). RC plants exhibited higher PAL activities with a significant increase between 1.16- and 1.39-fold (*P* ≤ 0.05) when compared with NIC plants during the entire time course tested (**Figure 4B**). In the ITC seedlings, the highest increase of 1.63-fold (*P* ≤ 0.05) at 72 h was evident when compared with that in the NIC plants (**Figure 4B**). Pathogen-inoculation significantly increased PAL activities in NIPI and RI plants, with the maximum of 3.01- and 2.39-fold (*P ≤* 0.05), respectively, at 48 hpi in relation to those in NIC and RC plants (**Figure 4B**). The time course analysis revealed a notable increase in PAL activities of ITPI plants from 12 hpi versus those of ITC plants, reaching a maximum increase of 2.01-fold (*P* ≤ 0.05) at 48 hpi (Supplementary Figure S1B). The ITC seedlings exhibited comparable PAL levels with those of RC at all time points (Supplementary Figure S1B). In addition, ITPI plants exhibited PAL activities comparable with those of RI plants at all time points (Supplementary Figure S1B).

Trehalose-induced POX activities were also analyzed across seven distinct time points in ITPI, ITC, NIPI, NIC, RI, and RC plants. We observed the maximum POX activity in ITC plants after 24 h, exhibiting a significant increase of 1.81-fold (*P* ≤ 0.05) over that of NIC plants (**Figure 4C**). Challenge-inoculation of pearl millet plants with the pathogen led to an increasing tendency in POX activities in a similar manner to those of PPO and PAL activities in ITPI plants versus ITC plants, with the maximum increase of 1.33-fold (*P* ≤ 0.05) observed at 24 hpi (Supplementary Figure S1C). Additionally, RI plants exhibited the highest increase of 2.08-fold (*P* ≤ 0.05) in POX activity at 24 hpi relative to that of RC plants (**Figure 4C**). POX activities were found to increase in ITC plants from 3 h, reaching a maximum at 24 h, then slightly reduced (Supplementary Figure S1C). We also noted that the POX activities were higher in ITC than RC seedlings at all the time points tested, with a highest increase of 1.63-fold (*P* ≤ 0.05) noted at 24 hpi in ITC plants (Supplementary Figure S1C). Under pathogen-inoculation, ITPI plants showed an increasing tendency in POX activities at levels comparable to those produced by RI plants (Supplementary Figure S1C).

## Discussion

Several promising alternative control strategies are based on the applications of natural resistance inducers. These inducers provide novel disease control methods that are not only environmentally benign but also help in understanding the complex mechanisms responsible for induced resistance (Trouvelot et al., 2014). Research into plant disease resistance has recently been focusing on sugar-mediated plant defense in order to discover the feasibility of using sugar-like compounds as substitutes to traditional agrochemicals in different cropping systems (Moghaddam and Van den Ende, 2012). For example, foliar application of 10 ppm glucose or fructose induced resistance in apple (*Malus domestica*) trees against western flower thrips, with remarkable increase in chlorogenic acid and trehalose levels in the leaves sprayed with either glucose or fructose (Derridj et al., 2009), suggesting that trehalose might contribute to enhanced plant defense responses against the thrips. The same authors also reported that foliar treatments of tomato (*Solanum lycopersicum*) with 100 ppm sucrose greatly reduced the disease symptom severity caused by *Botrytis cinerea* (Derridj et al., 2011). Additionally, supplementation of plant growth medium with 25 mM allose provided resistance to rice (*Oryza sativa*) seedlings against bacterial blight disease (Kano et al., 2011). In another independent study, exogenous seed treatment with trehalose resulted in an over-induction of wheat defense responses against *B. graminis* f.sp. *tritici*, and this induction of resistance was found to be correlated with the enhanced expression of pathogenesis-related genes (Tayeh et al., 2014). Therefore, the present investigation was an attempt to assess whether trehalose could activate the pearl millet defense systems against downy mildew, in addition to investigating its effects in promotion of plant growth.

Our data provided a strong evidence that trehalose treatment improved seed germination and seedling vigor of pearl millet (**Figures 1A,B**). Similar observation was reported by Horita and Saruyama (2006), wherein onion (*Allium cepa* L.) seeds treated with 400 mM trehalose stimulated a rapid and uniform seed germination under normal conditions. More recently, Akram et al. (2016) demonstrated that seed treatment with 25 mM trehalose significantly promoted radish (*Raphanus sativus* L.) fresh and dry biomasses, as well as chlorophyll *a* and total soluble sugar contents in the radish plants. These results potentiate the important roles of exogenous trehalose treatment in seed germination and seedling vigor under normal conditions. Additionally, our results showed that trehalose was not toxic to *S. graminicola*, as it showed no inhibition of *S. graminicola* sporangia production or zoospore release in an *in vitro* cytotoxic assay (**Figure 2**). The non-cytotoxic effect of trehalose on *S. graminicola* is an important characteristic to avoid the development of new races of the fungus with higher resistance to the induced resistance provided by the molecule. Reignault et al. (2001) also observed similar non-cytotoxic effect of trehalose on *B. graminis* f.sp. *tritici* that causes powdery mildew on wheat.

More importantly, we showed in the present study that trehalose could highly protect pearl millet against downy mildew under both greenhouse and field conditions (**Figures 3A,B**). Significant differences in the protection levels of pearl millet against downy mildew were found to be time- and dose-dependent. Under greenhouse conditions, the highest downy mildew disease protection level of 70.25% was recorded with 200 mM trehalose seed treatment for 9 h, while the shorter duration times with lower dose treatments exhibited lower protective effects against downy mildew (**Figure 3A**). This might be explained by the fact that trehalose is hydrophilic in nature (Tayeh et al., 2014). Furthermore, field studies performed under natural growing conditions showed that application of trehalose was effective with 67.25% disease protection (**Figure 3B**), which was almost similar to the protection level of Apron 35SD (72.35%), a frequently used commercial pesticide. Thus, our results suggest that trehalose could be effectively commercialized as an eco-friendly phytoprotectant for pearl millet downy mildew. It is well established that trehalose is extremely hydrophilic in nature. Thus, the extent of its penetration through the plant cuticle is important, which was supported by the finding that better protection was observed with higher concentrations and longer durations of trehalose treatments (**Figures 3A,B**). Our results also show that seed treatment with 200 mM trehalose for 9 h provided 70.25% disease protection, which was found to be the highest among all the concentrations (25, 50, 100, and 200 mM) and time durations (3, 6, and 9 h) tested under greenhouse conditions. Similar results were also observed under field conditions, when the highest concentration and longest duration of treatment (200 mM for 9 h) resulted in the maximum disease protection of 67.25%. This could be due to the fact that trehalose cannot very efficiently penetrate the hydrophobic plant cuticle; and therefore, treatments with more highly concentrated solutions for longer period or sequential applications could mitigate this obstacle (Reignault et al., 2001; Tayeh et al., 2014). Furthermore, trehalose showed no toxic effect on sporangial germination of *S. graminicola* (**Figure 2**), because there was no significant difference between trehalose and SDW treatment in either *in vitro* formation of sporangia or zoospore release. Therefore, trehalose might act as a protectant only and not as a curative in nature.

Seed treatment is an effective and economical way of controlling pearl millet downy mildew disease because pearl millet is mainly grown in low-input rain-fed agricultural system in which farmers cannot afford expensive and repeated control measures (Thakur et al., 2004; Zarafi et al., 2004; Murali et al., 2013). In recent years, increasing evidence has indicated that trehalose and its derivatives have the capacity to act as signal molecules that can induce plant resistance against various types of stress (Lunn et al., 2014; Mostofa et al., 2015). In the present study, it is interesting to note that trehalose seed treatment could effectively protect pearl millet against the downy mildew disease. Our results are in line with previously published reports in which the exogenous applications of trehalose have been shown to trigger resistance against powdery mildew in wheat (Reignault et al., 2001; Muchembled et al., 2006; Tayeh et al., 2014). Moreover, exogenous trehalose application enhanced resistance against green peach aphid in *Arabidopsis trehalose phosphate synthase11* (*tps11*) knockout mutant (Singh et al., 2011). Thus, our findings together with those of other labs firmly demonstrated that trehalose is an essential signaling molecule in plant defense responses against various pathogens.

Plants respond to the pathogen entry by activating defense mechanisms through coordinated alteration in gene expression (Zhang et al., 2009). Biotrophic pathogens like *S. graminicola* depend on the living plants for their nutrition. These pathogens intimately interact with the host cells to alter various metabolic processes to satisfy their needs (Glazebrook, 2005). However, in order to stop the pathogen at the site of entry, reactive oxygen species (ROS), namely superoxide anion ($O_{2}^{•–}$) and hydrogen peroxide (H_2_O_2_), are released at the site of pathogen ingress. Thus, ROS-based defense system effectively provides protection to plants against biotrophic pathogens. These oxygen intermediates act as signaling molecules and form part of a signaling cascade, thereby activating plant defense mechanisms (Doehlmann and Hemetsberger, 2013; Stam et al., 2014). The most commonly studied mechanism, which acts in the first line of defense, includes genes encoding POX, and PAL and PPO. However, the way these genes and corresponding enzymes react in terms of speed after pathogen infection is different, depending on the nature of plant-pathogen interactions (Jones and Dangl, 2006; Tsuda et al., 2009; Daudi et al., 2012). POX catalyzes the redox reaction of various substrates, leading to lignification and cross-linking of cell wall proteins. PAL converts *L*-phenylalanine to *trans*-cinnamic acid and ammonia, and thus being responsible for the synthesis of several defense-related secondary compounds like phenols and lignins (Almagro et al., 2009; Tahsili et al., 2014). PPOs are copper–metalloproteins that have been extensively studied for their roles in plant defense. Genes encoding PPOs and the PPO activities from various plants have been characterized in response to defense signals and induction of plant resistance (Nandeeshkumar et al., 2008; Doehlmann and Hemetsberger, 2013). Systemic acquired resistance (SAR) is responsible for priming the host defense mechanism in order to up-regulate expression of defense-related genes, leading to accumulation of PAL, PPO, POX, and other pathogenesis-related proteins in uninfected tissues to protect them against any imminent pathogen attack (Ramos Solano et al., 2008). Therefore, in order to ascertain whether trehalose treatment results in up-regulation of PAL, POX, and PPO leading to SAR, we have analyzed the activities of the above enzymes in our study. The relationship between enzymatic activities and phenotypic data strengthened our understanding of pearl millet resistance induced by trehalose treatment. Pearl millet plants raised from susceptible “HB3” seeds treated with 200 mM trehalose and challenged with *S. graminicola* ITPI plants resulted in higher activities of PPO, PAL, and POX than NIC and ITC plants (**Figure 4**; Supplementary Figures S1A–C). Furthermore, ITPI plants exhibited comparable PPO, PAL, and POX levels as those of RI plants (Supplementary Figures S1A–C). The defense mechanism mostly prevalent against biotrophic pathogens is the one involving the phenylpropanoid pathway, which results in synthesis of lignins and other secondary metabolites, such as phenolics, which contribute to preventing the pathogen at the site of infection (Doehlmann and Hemetsberger, 2013). These results together indicated the important role of trehalose in inducing these defense-responsive enzymes, thereby contributing to pearl millet defense response against downy mildew. Our findings are supported by a previous report that showed that exogenous trehalose treatment induced defense mechanism in wheat against powdery mildew by activating PAL and POX enzymatic activities (Reignault et al., 2001). Induced PPO, PAL, and POX activities can improve epidermal auto-fluorescence, preventing the further expansion of pathogen invasion in cereals (Motallebi et al., 2015). The auto-fluorescence is associated with papilla deposition, resulting in slowing down or even blocking fungal penetration (Maor and Shirasu, 2005; de Leon and Montesano, 2013). Enhanced activities of these enzymes also result in enhancement of signal transduction, preventing further pathogen spread (Kowalcyzk et al., 2004; Van Buyten and Höfte, 2013; Falcón-Rodríguez et al., 2014). PAL and POX are important components of phenylpropanoid pathway, and enhanced activities of these enzymes will lead to increased production of lignin and secondary metabolites, such as flavonoids and phenolic compounds, which have anti-microbial and anti-oxidant potential (Fraser and Chapple, 2011; Mahmoudi et al., 2016). Lignin, flavonoids, and phenolic compounds can inhibit pathogen penetration by increasing the rate of localized cell death, thereby preventing further pathogen spread from the sites of infection (Thatcher et al., 2005; Duan et al., 2014; Motallebi et al., 2015; Yoon et al., 2015). Additionally, both the timing and localization of increased PPO, PAL, and POX activities are important in preventing the pathogen infections (Nandeeshkumar et al., 2008). The enhancement of the activities of PAL, PPO, and POX by trehalose supports the hypothesis that trehalose could be responsible for the increased deposition of papillae. Since, the biotrophic pathogens spread easily from the site of infection, mechanisms like ROS production and development of host papillae also start immediately after pathogens or its elicitors are recognized. Once, the ROS or oxidative burst in biphasic manner is produced (Barna et al., 2012), associated downstream signaling pathways like phenylpropanoid pathway are also activated. Therefore, pathogen infection is likely to result in early activation of defense enzymes related to phenylpropanoid pathway, such as PAL, POX, and PPO (Spoel et al., 2007). Therefore, our finding on the early increase of enzyme activities between 3 and 24 h after pathogen infection supports this hypothesis. This state of enhanced physiological and biochemical readiness could be used by the host as a potential platform for further enhancement of oxidative and/or anti-oxidative defense in case of pathogen attack (Kawano, 2003; Van Buyten and Höfte, 2013).

Trehalose treatment activates SAR-like responses in *Arabidopsis* and wheat. Protein kinases and endo-1,4-β-D-glucanase have been found to increase in these plants when they were infected with the powdery mildew pathogen (Moghaddam and Van den Ende, 2012). Many other cellular responses leading to establishment of SAR have been identified using advanced molecular tools (Bae et al., 2005; Li and Tian, 2006; Trouvelot et al., 2014). The induction of defense-responsive mechanism and the physicochemical properties of trehalose, including the high structural stability under extreme temperature, and the strong ability to stabilize various biocompounds by inhibiting their irreversible denaturation under dehydration, are some of the unique features of trehalose (Iturriaga et al., 2009; Tayeh et al., 2014; Trouvelot et al., 2014). All these characters can promote the applications of trehalose as a useful principal component and stabilizer in development of agriculturally relevant commercial formulations for plant disease control.

## Author Contributions

SG, SJ, and HS conceived and designed the experiments. SG performed the experiments. SG, SJ, MA, and L-ST analyzed the data. HS contributed reagents/materials/analysis tools. SJ and MA prepared figures and graphs. SG, SJ, MA, and L-ST wrote the manuscript. All the authors read and approved the final manuscript. SG, SJ, MA, and L-ST revised the manuscript.

## Conflict of Interest Statement

The authors declare that the research was conducted in the absence of any commercial or financial relationships that could be construed as a potential conflict of interest.
