# Supplementary material for: Exogenous Trehalose Treatment Enhances the Activities of Defense-Related Enzymes and Triggers Resistance against Downy Mildew Disease of Pearl Millet
Source: Front Plant Sci. 2016 Nov 15;7:1593. doi: 10.3389/fpls.2016.01593 (PMC5109038; doi:10.3389/fpls.2016.01593)
Supplement: Supplementary file 1 [file Presentation_1.PDF]

*Supplementary Material*

# Exogenous trehalose treatment enhances the activities of defense-related enzymes and triggers resistance against downy mildew disease of pearl millet

Sharathchandra Ramasandra Govind, Sudisha Jogaiah, Mostafa Abdelrahman, Hunthrike Shekar Shetty and Lam-Son Phan Tran

***Correspondence:**

Sudisha Jogaiah (jsudish@kud.ac.in) and Lam-Son Phan Tran (sontran@tdt.edu.vn; son.tran@riken.jp)

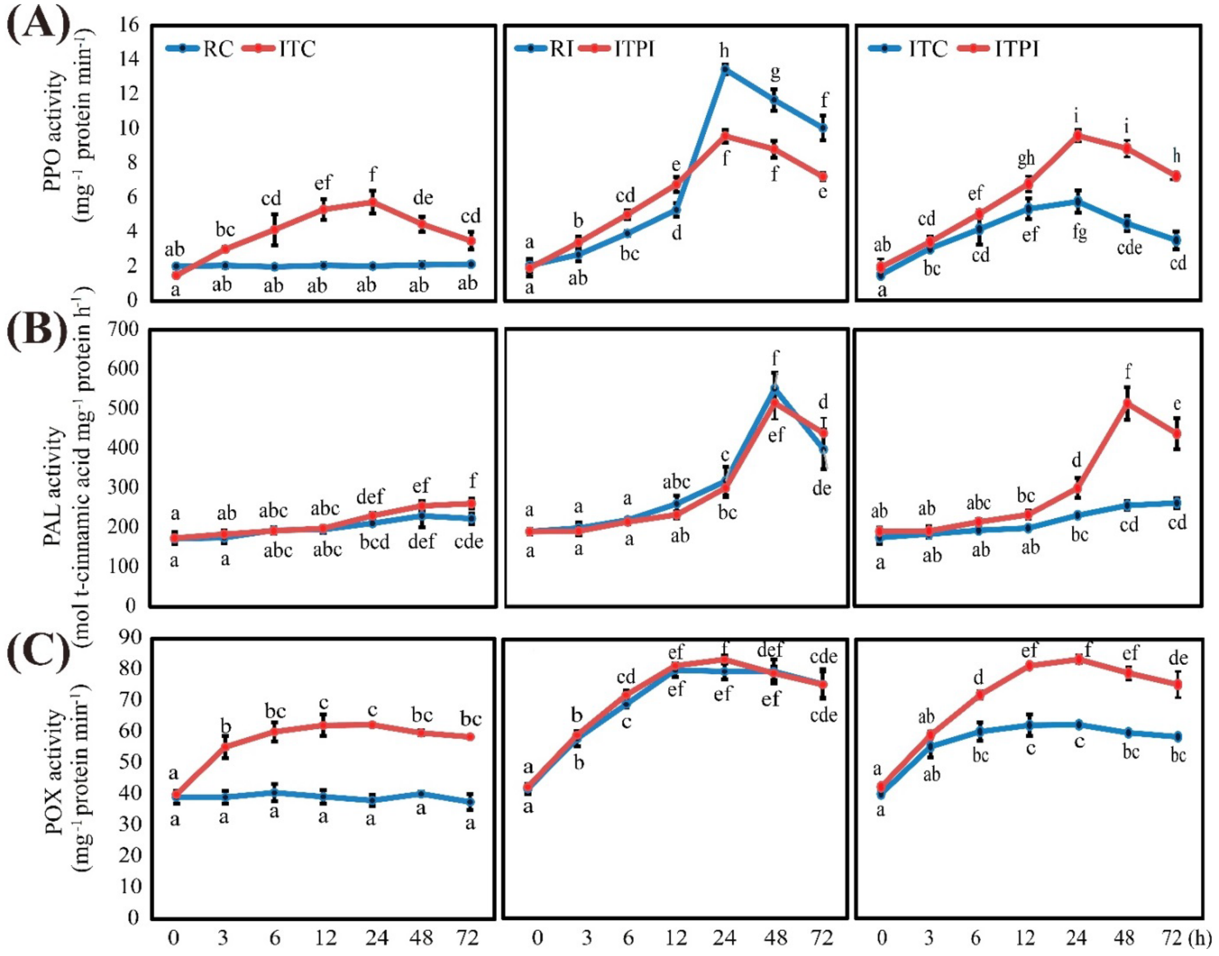


**Supplementary Figure S1. Temporal patterns of the polyphenol peroxidase (PPO), phenylalanine ammonia lyase (PAL) and peroxidase (POX) activities in 2-day-old pearl millet seedlings. (A)** PPO, **(B)** PAL and **(C)** POX activities from two-day-old susceptible “HB3” seedlings grown from seeds primed with sterile distilled water (SDW) or 200 mM trehalose for 9 h followed by root-dip inoculation with *Sclerospora graminicola* for various time periods (0, 3, 6, 12, 24, 48 and 72 h). “IP18192” seedlings grown from seeds primed with SDW for 9 h followed by root-dip inoculation with *S. graminicola* served as a positive control. Values are means ± standard errors (SEs) of four independent replicates ($n = 4$). Bars followed by different letters are significantly different according to a Tukey’s honestly significant difference (HSD) post hoc test ($P \leq 0.05$). Resistant control (RC), pearl millet resistant “IP18192” seedlings raised from seeds treated with SDW for 9 h and later challenged with SDW; resistant-inoculated (RI), pearl millet resistant “IP18192” seedlings raised from seeds treated with SDW for 9 h and later challenged with *S. graminicola*; inducer-treated control (ITC), susceptible pearl millet “HB3” seedlings raised from seeds treated with 200 mM trehalose for 9 h and later challenged with SDW; inducer-treated and pathogen-inoculated (ITPI), pearl millet susceptible “HB3” seedlings raised from seeds treated with 200 mM trehalose for 9 h and later challenged with *S. graminicola*.
